# Efficacy of vitamin D supplementation on glycemic control in type 2 diabetes patients: A meta-analysis of interventional studies

**DOI:** 10.1097/MD.0000000000014970

**Published:** 2019-04-05

**Authors:** Zhiwei Hu, Jin’an Chen, Xinjuan Sun, Lei Wang, Aiping Wang

**Affiliations:** Department of Endocrinology, 454 Hospital Affiliated to People's Liberation Army, Nanjing, Jiangsu Province, China.

**Keywords:** glycaemic control, meta-analysis, RCT, type 2 diabetes, vitamin D

## Abstract

**Background:**

Conflicting evidence exists on the effect of vitamin D supplementation on glucose metabolism in subjects with type 2 diabetes (T2D). Therefore, this meta-analysis focuses on the relationship between vitamin D intervention and glycaemic control in subjects with T2D.

**Methods:**

We reviewed available randomized controlled trials (RCTs) studies from the establishment time of each database to March 31, 2018. Stata 13.0 software was used to evaluate the included literature.

**Results:**

Finally, a total of 19 RCT studies involving 747 intervention subjects and 627 placebo controls were included in this meta-analysis. Meta-analysis results showed that compared with the control group, the short-term vitamin D supplementation group had a decline in hemoglobin A1c (HbA1c), insulin resistance, and insulin. The Standard Mean Difference (SMD) (95% CI [95% confidence interval]) of HbA1c, insulin resistance, and insulin were −0.17 (−0.29, −0.05), −0.75 (−0.97, −0.53), −0.57 (−0.78, −0.35), respectively with all *P* value <.05. But there were no significant differences in long-term follow-up vitamin D intervention.

**Conclusion:**

Vitamin D supplementation in T2D patients can improve HbA1c, insulin resistance, and insulin in short-term intervention, suggesting that vitamin D can be considered as a therapeutic agent along with the other treatments for T2D.

## Abstract 2

Supplemental Digital Content is available in the text

## 1 Introduction

Type 2 diabetes (T2D) is a public health challenge all over the world. By the end of 2015, a total of 392 million people suffer from T2D worldwide.^[1]^ T2D is a long-term metabolic disorder which is characterized by the relative lack of insulin, insulin resistance, and high blood glucose.^[2]^ It has been well known that T2D is a major risk factor for premature mortality and adverse complications such as blindness, stroke, heart attack, amputation, and kidney failure.^[3]^

Over the past few decades, series studies have evaluated the association of circulating Vitamin D concentrations with T2D risk and yielded a tight relationship between them, although the findings remain inconsistent.^[4]^ Scragg et al reported an inverse association between vitamin D status and diabetes,^[5]^ however no significant relationship of vitamin D status with fasting glucose and insulin was found in another cross-sectional study.^[6]^ Interestingly, 2 women nested case–control studies observed inconclusive results between plasma 25-(OH)D levels and risk of incident T2D.^[7,8]^ Numerous prospective cohort studies have demonstrated that higher vitamin D status was associated with decreased risk of T2D.^[9–11]^ These population epidemiological evidence indicated that Vitamin D plays an important role in T2D and sequent diseases. Therefore, the researches on the positive preventive effect of vitamin D supplement on diabetes were widely carried out.

Alcubierre et al suggested that vitamin D deficiency was significantly associated with lower quality of life, as well as with lower satisfaction with diabetes treatment.^[12]^ Recently, a randomized controlled trials (RCTs) study showed that vitamin D supplementation not only reduced blood glucose in T2D patients but also increased insulin sensitivity.^[13]^ Besides, it has also been suggested that the intake of vitamin D was inversely associated with the development of T2D complications.^[14]^ On the other hand, there was a lack of correlation between the use of vitamin D and insulin secretion rate neither and hemoglobin A1c (HbA1c) in T2D patients.^[15]^ RCTs studies reported a discrepancy between vitamin D supplementation and effeteness of T2D treatment. These inconsistent results might be partly due to small number of eligible participants. To systematically evaluate the effects of vitamin D supplementation on fasting blood glucose (FBG), insulin, HbA1c, and homeostasis model assessment-insulin resistance (HOMA-IR) in T2D patients, this RCT meta-analysis was conducted.

## 2 Materials and methods

Since this study is a meta-analysis of previously published studies, the ethical approval and patient consent are not required.

### 2.1 Search strategy

Comprehensive searches for eligible trials were performed by an electronic search of the PubMed, Elsevier database, Wiley database, Springer Link, and the Cochrane library. Searched the Title/Abstract using the following terms:(“Vitamin D” or “Cholecalciferol” or “calcitriol” or “Vitamin D2” or “Vitamin D3”) and (“Diabetes” or “T2D” or “hyperglycemia”). The time searched was from the establishment time of each database to March 31, 2018. All of the studies were limited to English language.

### 2.2 Eligibility and exclusion criteria

Eligibility criteria were set as follows:

(1) all studies had to be a RCT design;
(2) studies should provide at least one of the following outcomes (FBG or insulin or HbA1c or insulin resistance);
(3) insulin resistance estimated by HOMA-IR and HOMA-IR = (glucose, [mmol/L] x insulin [mU/L])/22.5^[16]^
(4) data description was mean ± SD;
(5) cases were T2D patients;
(6) the intervention group was treated with Vitamin D, while the control group was given placebo.

Exclusion criteria were set as follows:

(1) reviews, abstracts or animal studies;
(2) incompleteness of information data;
(3) error of statistical methods
(4) follow-up less than 2 months;
(5) cases had gestational diabetes, post partum diabetes, diabetic nephropathy, type 1 diabetes and high-risk population of diabetes.

### 2.3 Data collection

Two investigators independently scanned titles or abstracts to exclude studies which failed to meet the mentioned criteria and then obtained, reviewed and extracted the full-text reports for further assessment. Disagreements were resolved by consensus. Detailed data of eligible trials such as study design, participants’ information, methodological evaluation, intervention outcomes, and adverse event reports were extracted.

### 2.4 Statistical analysis

This meta-analysis adopted stata13.0 software for statistical analysis. SD (Δ) = [SD^2^ (baseline)+SD^2^ (final) −2 × 0.5 × SD (baseline)+SD^2^ (final)]^1/2^.^[17]^ Q test and the I^2^ index was used to assess the statistical heterogeneity. If there was no statistically significant heterogeneity (*P* >.1 and I^2^ <50%), a pooled effect was calculated with a fixed-effects model, whereas a random-effects model was employed on the contrary (*P* <.1 or I^2^ >50%). The strength of relationship between vitamin D supplementation and outcomes were by the value of the Standard Mean Difference (SMD) and 95% confidence interval (95%CI).

## 3 Results

### 3.1 Study characteristics

**Figure 1:**
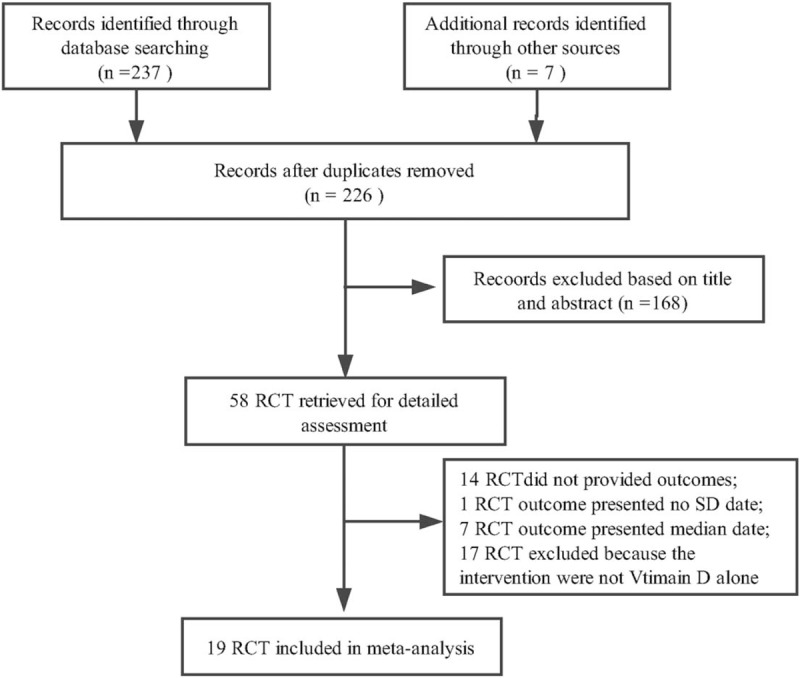
Flow chart of study selection.

In the primary search, 244 articles were retrieved, thereby 58 articles to further screening after reviewed on title and abstract. Finally, 19 independent studies accorded with conditions through full text reviewed.^[13,18–35]^ The detail omitted records were presented in the Figure 1. The study follow-up time with more than 6 months was considered as long-term study and those less than 6 months was considered as short-term study. All studies were used the JADAD scale to assessed the quality.^[36]^ Research in 3 points or more believed that the quality is high. The characteristics and scores of eligible studies were summarized in Supplement Table1.

### 3.2 Quantitative synthesis

In this meta-analysis, we did not observe a significant FBG change in vitamin D supplement intervention group. After a subgroup analysis by follow-up time, the result remained no statistical difference. The data was shown in Supplementary Figure 1.

**Figure 2:**
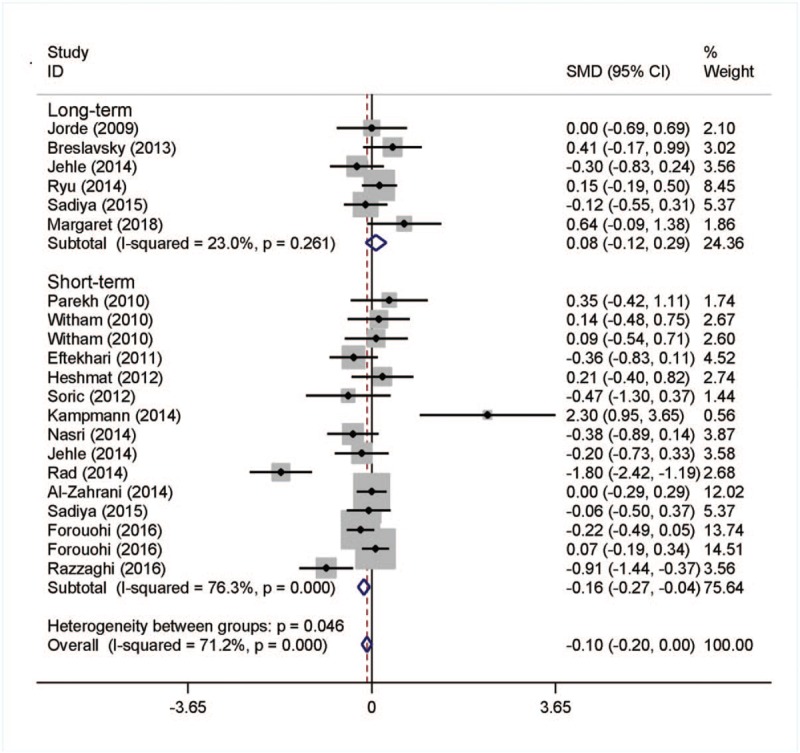
Meta-analysis of effects on HbA1c.

No significant difference was noted in HbA1c change between the intervention group and the placebo group. Whereas after a subgroup analysis, we found that the level of HbA1c decreased significantly in the short-term follow-up intervention group and the SMD (95% CI) was −0.17 (−0.29, −0.05) with *P* = .007. The data was shown in Figure 2.

**Figure 3:**
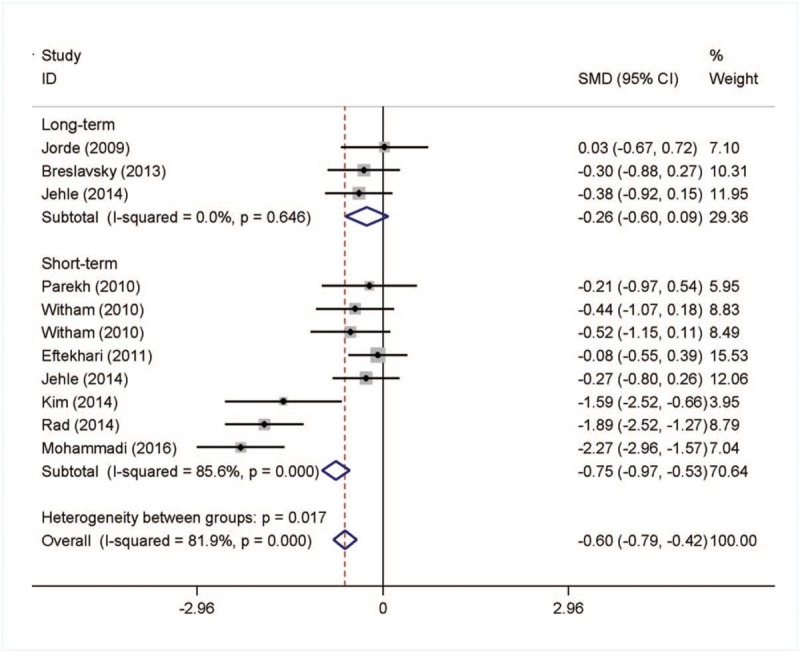
Meta-analysis of effects on insulin resistance.

In the 11 studies, there was significant decrease in HOMA-IR with vitamin D supplementation. In the subgroup analysis, we found that the level of HOMA-IR decreased significantly in the short-term follow-up intervention group and the SMD (95% CI) was −0.75 (−0.97, −0.53) with *P* <.001. The data was shown in Figure 3.

**Figure 4:**
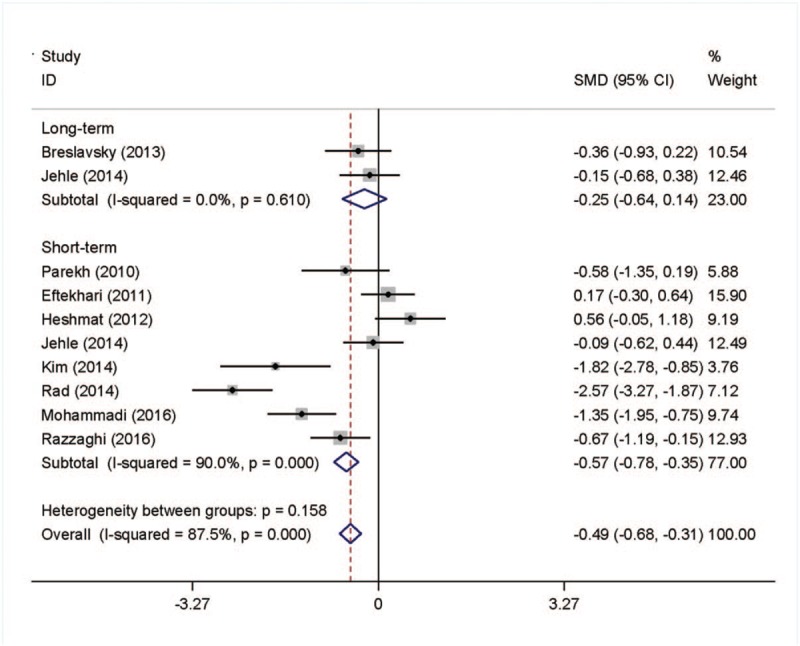
Meta-analysis of effects on insulin secretion.

Similarly, we observed insulin change in the vitamin D supplementation group. After subgroup analysis, we found that the level of insulin reduced significantly in the short-term follow-up vitamin D supplementation group and the SMD (95% CI) was −0.57 (−0.78, −0.35) with *P* <.001. The data was shown in Figure 4.

### 3.3 Publication bias and sensitivity analysis

Egger test was applied to assess the included literature publication bias in this study and no significant publication bias was found, the Egger test *P* value of the FBG, HbA1c, HOMA-IR, and insulin were 0.922, 0.450, 0.149, and 0.082, respectively.

Results showed high heterogeneity in HbA1c, HOMA-IR, and insulin except FBG (Figures 2–4, Supplementary Figure 1), then sensitivity analysis was carried out to find the sources of heterogeneity. After removing 1 study at a time, we found that the Rad et al study change the pooled HbA1c SMD quite big. The heterogeneity among the studies was clearly reduced (I^2^ = 57%) and the pooled SMD became no longer significant (*P* = .114).

Sensitivity analysis results demonstrated that most of the HOMA-IR heterogeneity belonged to the study of Rad et al and Mohammadi et al. The heterogeneity was reduced after exclusion of the 2 studies (I^2^ = 42%) and the pooled SMD remained significant.

## 4 Discussion

In this meta-analysis of prospective RCT designed for glycaemic control outcomes in subjects with T2D, we found that vitamin D supplementation prevented the increase in plasma HbA1c, insulin resistance and insulin in the subgroup of subjects with short-term follow-up intervention but had no apparent effect among those with long-term follow-up intervention. No effect of supplementation on plasma FBG among either subgroup was observed.

HbA1c is caused by continuous slow non-enzymatic glycosylation of hemoglobin due to hyperglycemia. The UK Prospective Diabetes Study demonstrated that HbA1c is a gold standard for the evaluation of glycemic control in the control of diabetes, with a 1% reduction, a 14% reduction in related cardiovascular events.^[37]^ George et al have conducted a meta-analysis to evaluate the effect of vitamin D on glycaemic control and insulin resistance.^[38]^ The paper found a small improvement on FBG and insulin resistance but no beneficial effect was seen on HbA1c. However, this meta-analysis included both impaired FBG patients and T2D patients. In our meta-analysis, we observed vitamin D supplementation prevented the increase in plasma HbA1c, suggesting that vitamin D is beneficial to reduce or delay the occurrence and development of diabetic complications. These inconsistent results might be due to our increased updated studies.

HOMA-IR was an important factor in the development of diabetes mellitus, and there was a significant positive correlation between them.^[39]^ The decreased sensitivity of insulin target tissues to insulin is called IR and most patients with T2D have combined IR.^[2]^ The level of blood glucose and insulin secretion can often reflect the sensitivity of insulin indirectly. At the same time, blood glucose has not been effectively controlled, which will further stimulate the β-cells to synthesize more insulin.^[40]^ Our results showed a significant decrease in insulin and HOMA-IR levels in vitamin D supplementation group. Insulin secretion is a calcium-dependent process. L-type calcium channels on islet β-cells are activated by activated vitamin D which then regulates calcium levels, initiates insulin signaling, and promotes insulin release.^[41]^ Vitamin D deficiency can be accompanied by a decrease in plasma calcium concentration, which in turn causes a secondary increase in calcium levels, affecting insulin signal transduction, interfering with insulin release and disrupting islet β cell function.^[42]^ Fatty acid metabolism in skeletal muscle and adipose tissue is regulated by peroxisome proliferator-activated receptor (PPAR-δ) and PPAR-δ has a certain regulatory effect on IR. Vitamin D can directly activate PPAR-δ expression, thereby improving IR.^[43]^

Studies have shown that vitamin D deficiency is associated with the development of T2D, T2D nephropathy, T2D microvascular or macrovascular disease, diabetic retinopathy, and diabetic peripheral neuropathy.^[44–48]^ In an 11-year cohort follow-up study, baseline vitamin D levels were negatively correlated with the development of T2D.^[49]^ The genetic polymorphisms of vitamin D binding protein and vitamin D receptor are associated with genetic susceptibility to T2D. In a Japanese study, significant differences in insulin and IR between different vitamin D binding protein genotypes were found in adults with normal glucose tolerance.^[50]^ Therefore, the present study further supports these work and indicate that vitamin D supplementation improves IR in patients with T2D in the short term.

In the subgroup analysis, there was no apparent decreased in plasma HbA1c, HOMA-IR and insulin with long-term follow-up intervention. This may be due to random errors in the study itself. On the other hand, the fact that the prolonged duration of T2D or gradually worsen with the course of T2D may help to explain the result.^[51]^ Moreover, it has been shown that compared with FBG, the 2 hours postprandial blood glucose contributes more to HbAlc,^[52]^ which may be an important reason for the difference HbAlc between vitamin D supplementation group and the control group, but no statistical significance in FBG.

Sensitivity analysis suggested that the major sources of heterogeneity in results were due to the studies of Rad et al^[30]^ and Mohammadi et al.^[33]^ The other causes such as ethnic, the health status of patients and quality of the studies may lead to the heterogeneity.

There are some limitations in this study. First, the long-term follow-up studies included are few. Second, we did not consider the vitamin D intake dose variations in the include studies which may generate some heterogeneity and the inconsistency in the supplemental dose of vitamin D may cause a certain shift in the combined values. Finally, although the heterogeneity of the literature was circumvented, heterogeneity still existed. Of course, based on the perspective of evidence-based medicine, this study shows that vitamin D supplementation can play an important role in controlling blood glucose and improving IR.

In summary, vitamin D supplementation in T2D patients can improve HbA1c, insulin resistance, and insulin in short-term intervention, but the impact on the FBG is not significant. Therefore, vitamin D can be considered as a therapeutic agent along with the other treatments for T2D.

## Author contributions

**Conceptualization:** Aiping Wang.

**Data curation:** Jinan Chen, Lei Wang.

**Methodology:** Xinjuan Sun.

**Writing – original draft:** Zhiwei Hu.

## Supplementary Material

Supplemental Digital Content
