# Supplementary material for: Efficacy of vitamin D supplementation on glycemic control in type 2 diabetes patients: A meta-analysis of interventional studies
Source: Medicine (Baltimore). 2019 Apr 5;98(14):e14970. doi: 10.1097/MD.0000000000014970 (PMC6456062; doi:10.1097/MD.0000000000014970)

| Supplement Table 1. Summary of abstracted articles in meta-analysis | | | | | | | | |
| --- | --- | --- | --- | --- | --- | --- | --- | --- |
| Study | Author | Year | Country | N(control/Intervention) | Intervention(1) | Intervention(2) | Duration | JADAD score |
| 1 | Jorde | 2009 | Norway | 16/16 | 40000IU,weekly |  | 6 months | 4 |
| 2 | Parekh | 2010 | India | 13/14 | 300000IU,single IM |  | 4 weeks | 3 |
| 3 | Witham | 2010 | UK | 22/19/18 | 200000IU,single | 100000IU,single | 16 weeks | 5 |
| 4 | Eftekhari | 2011 | Iran | 35/35 | 5000IU,daily |  | 12 weeks | 3 |
| 5 | Heshmat | 2012 | Iran | 21/21 | 300000IU ,single IM |  | 12 weeks | 3 |
| 6 | Soric | 2012 | USA | 8/19 | 2000IU,daily |  | 12 weeks | 2 |
| 7 | Breslavsky | 2013 | Israel | 23/24 | 1000IU,daily |  | 12 months | 3 |
| 8 | Kampmann | 2014 | Danish | 8/7 | 11200IU daily for 2weeks; then 5600IU daily for 10weeks |  | 12 weeks | 4 |
| 9 | Nasri | 2014 | Iran | 30/30 | 50000IU,weekly |  | 12 weeks | 3 |
| 10 | Jehle | 2014 | Switzerland | 26/29 | 150000IU,single IM |  | 6 months | 5 |
| 11 | Kim | 2014 | Korea | 13/11 | 1200IU,daily |  | 12 weeks | 0 |
| 12 | Ryu | 2014 | Korea | 65/64 | 2000IU,daily |  | 6 months | 4 |
| 13 | Rad | 2014 | Iran | 30/28 | 4000IU,daily |  | 8 weeks | 3 |
| 14 | Al-Zahrani | 2014 | Saudi | 92/91 | 45000IU weekly for 8weeks,then 45000IU single for 4weeks |  | 12 weeks | 3 |
| 15 | Sadiya | 2015 | UAE | 43/39 | 6000IU daily for 3months,then 6000IU daily for 3months, |  | 6 months | 5 |
| 16 | Mohammadi | 2016 | Iran | 25/28 | 50000IU,weekly |  | 12 weeks | 3 |
| 17 | Forouohi | 2016 | UK | 111/100/110 | 20000IU VitD2, daily | 20000IU VitD3, daily | 4 months | 4 |
| 18 | Razzaghi | 2016 | Iran | 30/30 | 50000IU,twice weekly |  | 12 weeks | 3 |
| 19 | Margaret | 2018 | USA | 16/14 | 50000IU,weekly |  | 6 months | 3 |


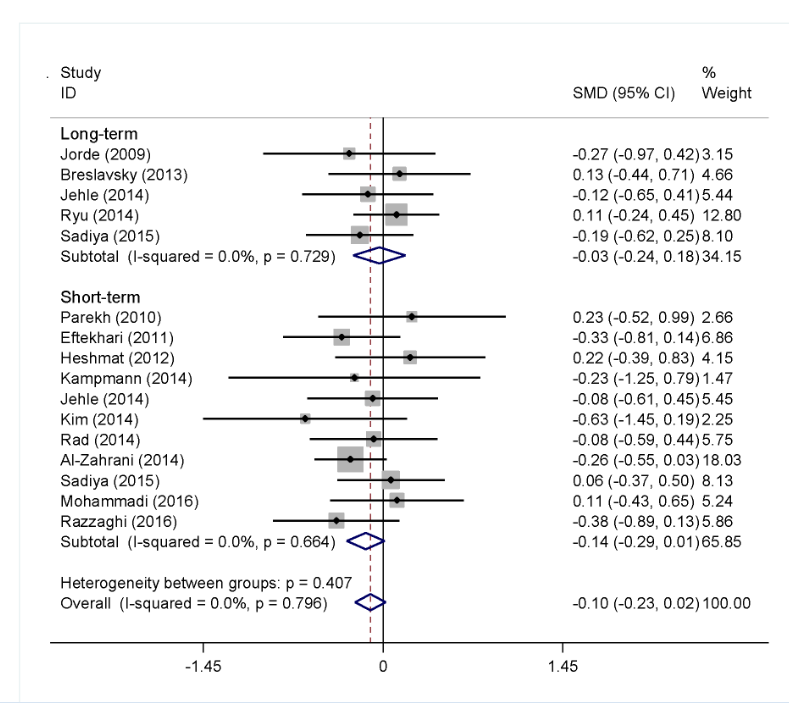

Supplement: Supplemental Digital Content [file medi-98-e14970-s001.doc]
